# Insights into the airborne microorganisms in a Sichuan south-road dark tea pile fermentation plant during production

**DOI:** 10.3389/fmicb.2024.1439133

**Published:** 2024-09-02

**Authors:** Miaoyi Liu, Xian Li, Yimiao Li, Yao Zou

**Affiliations:** ^1^Department of Tea Science, College of Horticulture, Sichuan Agricultural University, Chengdu, China; ^2^Tea Refining and Innovation Key Laboratory of Sichuan Province, Chengdu, China

**Keywords:** Sichuan south-road dark tea, pile fermentation plant, airborne microorganism, microbial community structure, function

## Abstract

**Introduction:**

Sichuan south-road dark tea (SSDT) is generally produced through a series of processes, including fixing, rolling, pile fermentation, and drying, with microbial action during pile fermentation playing a crucial role in determining tea quality. The air within the SSDT pile fermentation plant (SSDTPP) is considered an important source of these microbes, but research in this area has been limited.

**Methods:**

In this study, air samples from SSDTPP were collected on the 1st (SSDT1), 12th (SSDT2), and 24th (SSDT3) days of pile fermentation and comprehensively analyzed by high-throughput sequencing.

**Results and discussion:**

The results revealed the presence of 2 and 24 phyla, 9 and 49 classes, 18 and 88 orders, 28 and 153 families, 38 and 253 genera, and 47 and 90 species of fungi and bacteria, respectively, across all samples. SSDT1 and SSDT2 individually had the highest fungal and bacterial diversity, while *Aspergillus* was the dominant genus throughout the pile fermentation with an abundance of 34.6%, 91.17%, and 67.86% in SSDT1, SSDT2, and SSDT3, respectively. Microbial populations in SSDT1 were predominantly involved in xenobiotic biodegradation and metabolism, amino acid metabolism, the biosynthesis of other secondary metabolites, etc. However, SSDT2 exhibited a higher prevalence of human disease-related functions. SSDT3 primarily focused on the metabolism of other amino acids and carbohydrate metabolism. Additionally, 104 genera and 22 species coexisted in both SSDTPP air and piled SSDT, suggesting that frequent microbial exchange may occur between them. These findings pave the way for microbial traceability during SSDT production and provide a foundation for further functional microbial research.

## Introduction

Tea is the most famous beverage in China. It can be classified into six types, namely, green tea, yellow tea, dark tea, white tea, oolong tea, and black tea, according to their production process and distinctive flavor (Tang et al., 2019). In recent years, dark tea has attracted increasing attention due to its excellent hypolipidemic and hypoglycemic properties, ability to relieve greasiness, and weight loss benefits (Chen et al., 2018; Xiao et al., 2020; Wu et al., 2023). Historically, it was mainly sold to the country's border regions as a vital source of sustenance for the Indigenous ethnic minorities (Wang et al., 2016). Hence, it is also known as “border-selling tea.” Chinese dark tea has typical terroir characteristics, and the tea leaves produced in the provinces of Sichuan, Hunan, Yunnan, Hubei, and Guangxi have different flavors (Lin et al., 2021). Sichuan south-road dark tea (SSDT) is the main type of Sichuan dark tea (SDT) and the bulk of China's dark teas (Supplementary Figure S1). It is generally pressed into bricks with a bright yellow-red broth color, mellow taste, and pure and aged tea aroma (Zou et al., 2022).

Pile fermentation is a crucial process in dark tea production that exposes the tea to the air, allowing for the accumulation of airborne microorganisms, which accelerates the formation of dark tea quality. During this process, *Aspergillius* species play a significant role in the hydrolysis, oxidation, polymerization, and degradation of phenolic compounds in Pu'erh tea (Ma et al., 2021). *Eurotium cristatum* contributes to the development of Fu-brick tea quality by increasing levels of volatile organic compounds that impart stale and floral aromas (Zhu et al., 2020; Wang et al., 2021; Xiao et al., 2022), while *Debaryomyces hansenii* is heavily involved in carbohydrate degradation and catechin conversion of SSDT (Zou et al., 2023a).

Indeed, the flavor development in many traditional fermented foods is strongly related to the local microbial community and environmental factors (Li et al., 2022). The concept of “microbial terroir,” which includes region, site specificity, air temperature, humidity, and other environmental factors, significantly influences the geographic distribution of microorganisms. When combined with the fermentation microenvironment of piled tea, these factors can greatly help shape the microbial communities in piled dark tea, ensuring the “one place, one flavor” characteristic that is unique to dark tea from different regions.

In the pile fermentation plant, microbes inhabiting piled dark tea usually come from the surrounding air, manufacturing tools, running water, workers, etc (Zhang et al., 2013). Airborne microbes can be electrostatic and adhere to the surface of small particles, which means they can be easily transported by the wind to different locations and deposited on the surface of any object (Jahne et al., 2015; Zickrick et al., 1995). Therefore, the pile fermentation plant air has always been considered the primary microbial source of piled tea. Recently, many studies have investigated the microorganisms involved in dark tea pile fermentation, focusing on microbial communities, dominant microbial populations, and microbial functions (Zou et al., 2022, 2023b; Li et al., 2018). However, only a handful of studies have focused on airborne microbes involved in pile fermentation during production.

This study aimed to characterize the airborne microbial community of a typical SSDTPP during pile fermentation using a non-culture method combined with high-throughput sequencing and elucidate the microbial community function. The results will serve as a valuable reference for microbial traceability of piled SSDT and ensure its safe production. It will also provide important functional microbial information for dark tea production and contribute to understanding the geographical uniqueness of dark tea quality.

## Materials and methods

### Sampling

The sampling site was set up at a representative SSDTPP in Ya'an City, Sichuan Province, China (29°56′N, 103°7′E). Sampling was conducted on the 1st, 12th, and 24th days of pile fermentation during the peak production period in August 2018, with three parallel samples collected at each sampling time. Airborne microorganisms from the SSDTPP were captured by a total suspended particle sampler (Tianhong, TH-150, Wuhan, China) at a height of 50 cm above the surface of piled tea with continuous collection on quartz membranes (90 mm, Whatman) at an average flow rate of 100 L/min. The membranes were removed from the sampler and stored at −20°C for subsequent analysis. Sampling was performed between 9 and 11 a.m., before which the membrane holders were cleaned with a 75% ethanol solution. The pile fermentation lasted for 24 days, during which the air temperature and relative humidity were kept at 28–35°C and 70%−85%, respectively, while the temperature and water content of the piled tea were controlled at 35–70°C and 15%−30%, respectively.

### DNA extraction

The membranes were cut, then immersed in sterile ddH_2_O, oscillated for 30 min, followed by differential centrifugation, and the precipitate was used for DNA extraction. Microbial genomic DNA was extracted using the DNeasy^®^ PowerSoil^®^ Kit (QIAGEN, Germany) according to the manufacturer's instructions. After checking its concentration and integrity by spectrophotometric quantification and electrophoresis on a 1.0% agarose gel, respectively, the V3 and V4 regions of the bacterial 16S rRNA were amplified by PCR using the 515F (341F) and 907R (806R) primers according to the procedure described by Wang et al. (2018), while the ITS1 region of the fungal ITS rDNA was amplified using the primers of ITS1F and ITS2R according to the procedure described by Li et al. (2017). Amplification was conducted in triplicate. PCR products were then extracted, purified, pooled, and used for sequencing.

### Sequencing and phylogenetic analysis

Sequencing was performed on the Illumina MiSeq platform from Basebio Co., Ltd. (Chengdu, China). The QIIME program (version 1.9.1) was employed to de-multiplex and filter the raw data. UPARSE (version 7.1 http://drive5.com/uparse/) was used for operational taxonomic units (OTUs) clustering at 97% similarity. Chimeric sequences were identified and removed using UCHIME. Taxonomy was assigned to each representative sequence utilizing the RDP classifier (http://rdp.cme.msu.edu/) against the SILVA database (http://www.arb-silva.de) with a confidence threshold of 80% (Han et al., 2018).

### Functional annotation and microbial comparative analysis

FUNGuild (https://www.bioincloud.tech/standalone-task-ui/funguild) (Gao et al., 2024) and Tax4Fun (Aßhauer et al., 2015) were employed to predict the fungal and bacterial functions, respectively. Moreover, an additional comparative analysis of microbes in SSDTPP air and piled SSDT was conducted using the microbial data of piled tea collected at the same SSDTPP and production time (data were found at https://www.ncbi.nlm.nih.gov/, PRJNA828626) (Zou et al., 2022).

### Statistical analysis

The alpha diversity index of microbial communities in different air samples was calculated using Mothur (Schloss et al., 2009). The R software 3.5.0 (R Core Team, Vienna, Austria) was adopted to generate the histograms of microbial communities. Origin 2021 (Origin Lab Corporation, MA, United States) was utilized to construct the Sankey, Chord, and Lollipop diagrams. TBtools-II (Chen et al., 2023) was used to generate the bubble heatmap.

## Results

### Overview of airborne microbial sequencing data

Illumina high-throughput sequencing was performed on the SSDTPP airborne microorganism. After eliminating incomplete and poor-quality reads, a total of 154,031 high-quality fungal genomic sequences were obtained, including 31,833 sequences in SSDT1, 33,724 sequences in SSDT2, and 88,474 sequences in SSDT3, with read lengths ranging from 100 to 456 bp. Meanwhile, 120,464 high-quality bacterial genomic sequences were also observed, consisting of 46,136 sequences in SSDT1, 39,990 sequences in SSDT2, and 34,338 sequences in SSDT3, with read lengths ranging from 100 to 460 bp (Supplementary Table S1; Supplementary Figures S2A, B). Moreover, the rank-abundance curves of fungal OTUs for SSDT1 and SSDT3 were wider and flatter than that of SSDT2, indicating their higher richness and uniformity of fungal species, whereas SSDT2 showed a relatively steep declining curve, indicating its lower fungal diversity but higher proportion of dominant fungal species (Supplementary Figure S2C). Similarly, SSDT2 and SSDT3 had higher bacterial species richness and uniformity than SSDT1, while SSDT1 had lower bacterial diversity but a higher proportion of dominant bacterial species (Supplementary Figure S2D).

### Operational taxonomic unit cluster analysis

**Figure 1:**
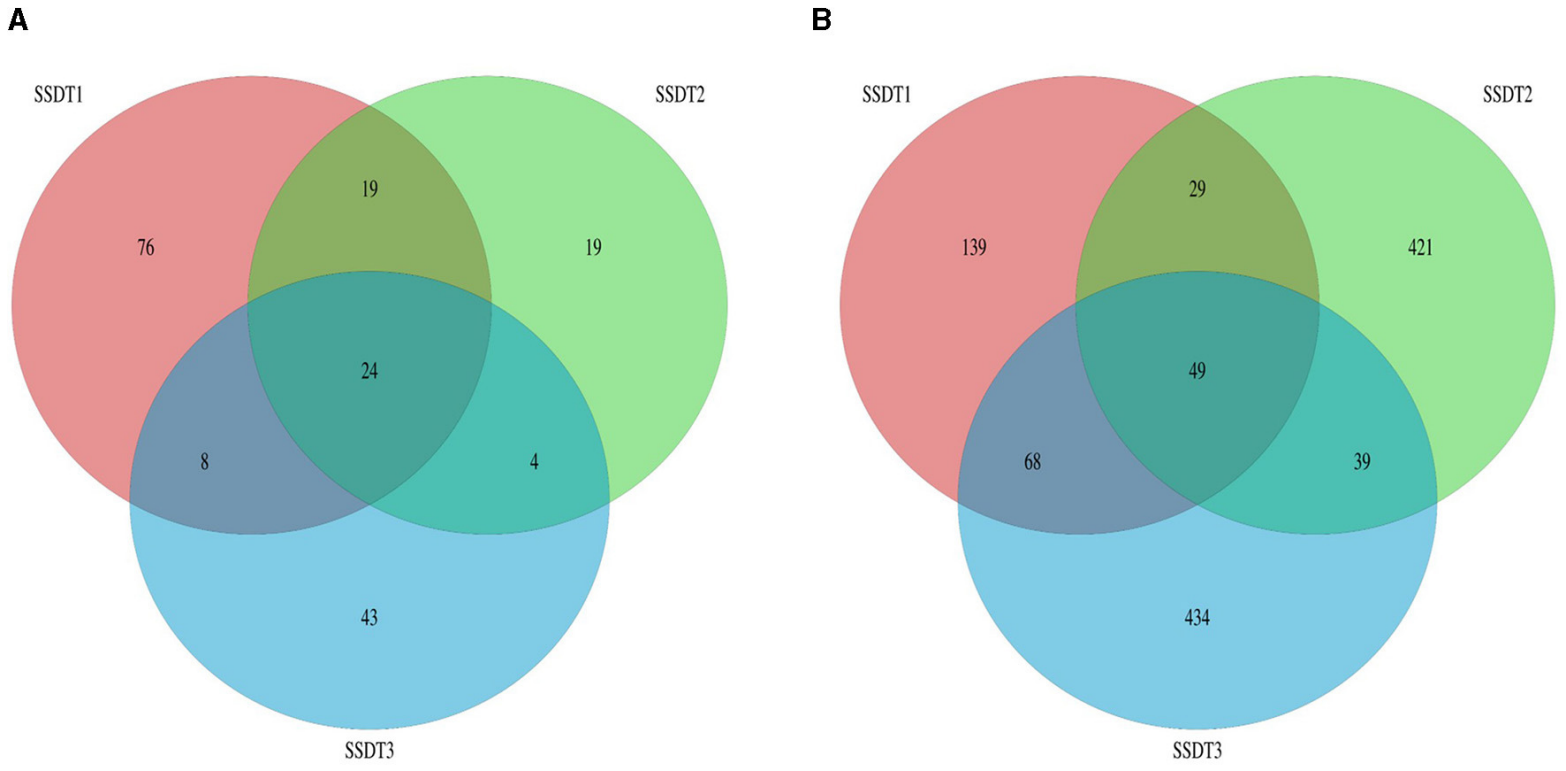
Venn analysis of OTU, different colors indicate the different samples and the numbers reflect the OTUs. **(A)** Fungal OTU. **(B)** Bacterial OTU.

Following the separation of non-repetitive single sequences from the optimized sequences and the elimination of redundant sequences, an OTU clustering analysis of the non-repetitive sequences was conducted with a 97% identity threshold. Finally, 193 fungal OTUs were observed, which were grouped into two phyla, nine classes, 18 orders, 28 families, 38 genera, and 47 species, with SSDT1 exhibiting the most abundant classified OTU information, for which 105, 86, 78, 74, 61, and 43 OTUs were grouped into phylum, class, order, family, genus, and species, respectively (Table 1). Only 24 fungal OTUs were common to all samples, while 76, 19, and 43 OTUs were unique to SSDT1, SSDT2, and SSDT3, respectively, while 43 fungal OTUs were shared by SSDT1 and SSDT2, 32 OTUs were shared by SSDT1 and SSDT3, and 28 OTUs were shared by SSDT2 and SSDT3, respectively (Figure 1A). Furthermore, a total of 1,179 bacterial OTUs classified into 24 phyla, 49 classes, 88 orders, 153 families, 253 genera, and 90 species were discovered, and SSDT3 presented the richest information of OTUs classified, with 588, 584, 551, 517, 405, and 65 OTUs individually grouped into phylum, class, order, family, genus, and species (Table 1). Although 49 bacterial OTUs were common to all samples, 139, 421, and 434 OTUs were unique to SSDT1, SSDT2, and SSDT3, respectively. Moreover, 78 OTUs were common between SSDT1 and SSDT2, 117 OTUs were common between SSDT1 and SSDT3, and 88 OTUs were common between SSDT2 and SSDT3 (Figure 1B).

**Table 1:** OTU classification information of airborne microbes in SSDTPP during pile fermentation.

**Sample**	**Phylum**		**Class**		**Order**		**Family**		**Genus**		**Species**	
	**Fungi**	**Bacterium**	**Fungi**	**Bacterium**	**Fungi**	**Bacterium**	**Fungi**	**Bacterium**	**Fungi**	**Bacterium**	**Fungi**	**Bacterium**
SSDT1	105	285	86	282	78	276	74	265	61	222	43	36
SSDT2	61	534	53	528	46	511	42	462	34	360	25	35
SSDT3	73	588	62	584	57	551	51	517	44	405	31	65
Total	2	24	9	49	18	88	28	153	38	253	47	90

The numbers in the last row (Total) represent the number of microbes at each classification level, and the numbers in the other rows represent the number of OTUs classified at each classification level.

### Airborne microbial diversity of SSDTPP

ACE and Chao1 indices typically reflect the richness of microbial populations. In this study, SSDT1 and SSDT2 showed the highest and lowest fungal richness with ACE values of 127.60 and 71.63 and Chao1 values of 130.00 and 73.20, respectively. Meanwhile, the highest and lowest bacterial richness was observed in SSDT3 and SSDT1, with ACE values of 594.92 and 288.73 and Chao1 values of 624.00 and 288.27, respectively. The Shannon and Simpson's indices usually reflect the diversity of microbial communities. In this study, SSDT1 and SSDT2 showed the highest and lowest fungal community diversity, with the Shannon index of 3.99 and 0.63 and the Simpson index of 0.87 and 0.18, respectively, while the highest and lowest bacterial community diversity was discovered in SSDT2 and SSDT1, with the Shannon index of 8.07 and 5.67, and the Simpson index of 0.99 and 0.93, respectively. Apart from that, the coverage of all samples was >0.999, indicating that the sequencing results could accurately represent the actual microbial composition of the air samples (Table 2).

**Table 2:** Alpha diversity analysis of the airborne microbial community in SSDTPP air during pile fermentation.

**Sample**	**Coverage**		**Ace**		**Chao1**		**Shannon**		**Simpson**	
	**Fungi**	**Bacterium**	**Fungi**	**Bacterium**	**Fungi**	**Bacterium**	**Fungi**	**Bacterium**	**Fungi**	**Bacterium**
SSDT1	0.999906	0.999804	127.60	288.73	130.00	288.27	3.99	5.67	0.87	0.93
SSDT2	0.999898	0.999550	71.63	545.72	73.20	559.86	0.63	8.07	0.18	0.99
SSDT3	0.999911	0.999505	80.46	594.92	80.50	624.00	2.93	7.93	0.67	0.99

### Dynamic changes in SSDTPP airborne microbial communities during production

**Figure 2:**
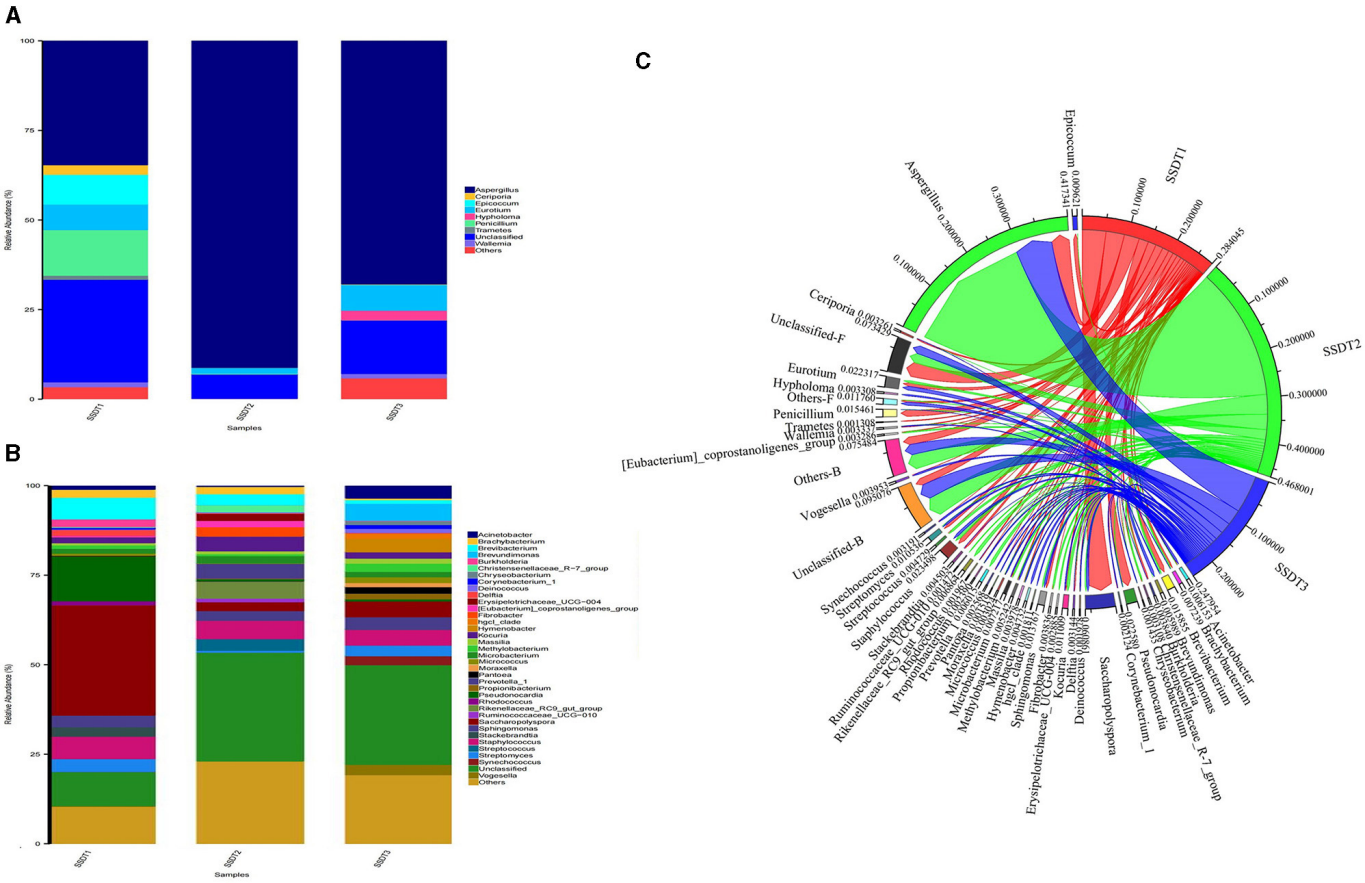
Changes in the microbial communities of SSDTPP air during pile fermentation. **(A)** The fungal community structure at the genus level, different genera are represented by columns with different colors, the length of each column indicates the size and proportion of the genus. **(B)** The bacterial community structure at the genus level. **(C)** Dynamic changes of airborne microbial populations at different fermentation stages at the genus level, the sectors with different colors represent the samples and the microorganisms, and the sector width reflects the relative abundance of microbes, with its value indicated by the number next to the sector.

A remarkable change in the microbial community of SSDTPP air was observed at the different stages of pile fermentation. It was found that for the fungal communities, *Aspergillus* was dominant in all samples, with an abundance of 34.6%, 91.17%, and 67.86% in SSDT1, SSDT2, and SSDT3, respectively. *Penicillium* (12.73%), *Epicoccum* (8.28%), *Eurotium* (7.15%), *Ceriporia* (2.67%), *Wallemia* (1.37%), and *Tramates* (1.13%) were also abundant in SSDT1. *Eurotium* (1.61%), *Penicillium* (0.21%), and *Wallemia* (0.08%) existed in SSDT2, while *Eurotium* (7.20%) showed a higher abundance in SSDT3, followed by *Hypholoma* (2.69%), *Wallemia* (1.23%), *Ceriporia* (0.13%), and *Penicillium* (0.02%); Figure 2A). In terms of bacterial communities, *Saccharopolyspora* (30.80%), *Peseudonocardia* (12.46%), *Brevibacterium* (6.02%), and *Burkholderia* (2.14%) were dominant in SSDT1. *Staphylococcus* (5.15%), *Kocuria* (4.14%), *Prevotella* (4.13%), and *Brevibacterium* (3.07%) also showed a relatively higher abundance in SSDT2, while *Brevundimonas* (4.77%), *Saccharopolyspora* (4.39%), and *Hymenobacter* (3.78%) were relatively abundant in SSDT3 (Figure 2B). Additionally, the total microbial abundance showed a trend of SSDT2 > SSDT1 > SSDT3 during pile fermentation, and the majority of microorganisms were present throughout the pile fermentation process, with *Aspergillus* being the most abundant at each fermentation stage (Figure 2C).

### Microbial comparative analysis

**Figure 3:**
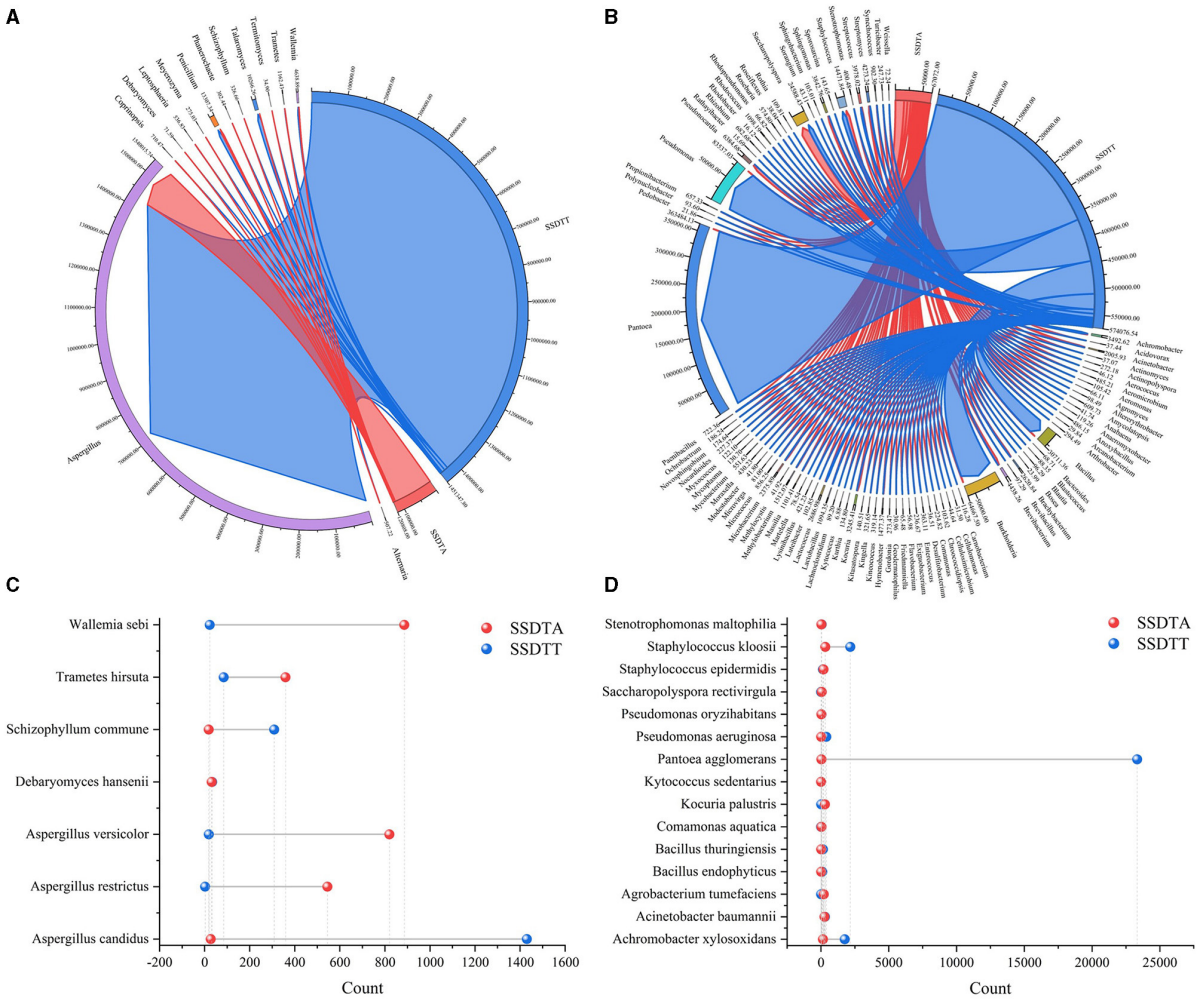
Comparative analysis of microbes in SSDTPP air and piled SSDT. **(A)** Fungal comparison at the genus level, SSDTA represents the microbes in SSDTPP air, and SSDTT represents the microbes in piled SSDT; the sector width represents the microbial abundance with the value indicated by the number next to the sector. **(B)** Bacterial comparison at the genus level, the sector width value represents the microbial abundance. **(C)** Fungal comparison at the species level, the vertical axis represents the microbial name, and the horizontal axis represents the microbial numbers; the colored dot represents the microbes in SSDPP air or piled SSDT. **(D)** Bacterial comparison at the species level.

The comparative analysis for microbes in SSDTPP air and piled SSDT revealed that a total of 104 genera, including *Aspergillus, Penicillium, Debaryomyces, Leptosphaeria, Meyerozyma, Schizophyllum, Saccharopolyspora, Achromobacter, Bacillus, Burkholderia, Phanerochaete, Streptomyces*, etc., and 22 species, such as *Aspergillus candidus, Aspergillus restrictus, Debaryomyces hansenii, Wallemia sebi, Pantoea agglomerans, Acinetobacter baumannii*, and *Staphylococcus epidermidis*, coexisted in both piled SSDT and SSDTPP air (Figure 3), among which, except for *Aspergillus*, most fungi showed higher abundance in SSDTPP air and most bacteria were abundant in piled tea. *Aspergillus* was strikingly dominant in both, with a markedly higher abundance in piled tea than in the air (Figures 3A, B). *Talaromyces, Wallemia, Achromobacter, Bacillus, Burkholderia, Pseudomonas, Schizophyllum commune, Pantoea agglomerans, Staphylococcus kloosii*, etc. also were present in a higher abundance in piled tea than in the air (Figure 3). Conversely, *Pseudonocardia, Streptomyces, Wallemia sebi, Aspergillus versicolor*, etc., were more abundant in SSDTPP air (Figures 3B, C). Furthermore, *Debaryomyces hansenii* and most of the coexisting bacteria species, such as *Acinetobacter baumannii, Staphylococcus epidermidis*, and *Pseudomonas oryzihabitans*, showed similar abundance in both SSDTPP air and piled tea (Figures 3C, D).

### Airborne microbial function

To elucidate the functional composition of microbial communities in SSDTPP air during pile fermentation, Taxa4Fun and FUNGuild were individually employed to predict the bacterial and fungal functions, respectively. It was found that the bacterial community in SSDT1 exhibited a dominant function in xenobiotics biodegradation and metabolism, lipid metabolism, metabolism of terpenoids and polyketides, amino acid metabolism, biosynthesis of other secondary metabolites, transport and catabolism, endocrine system and excretory system, etc.

**Figure 4:**
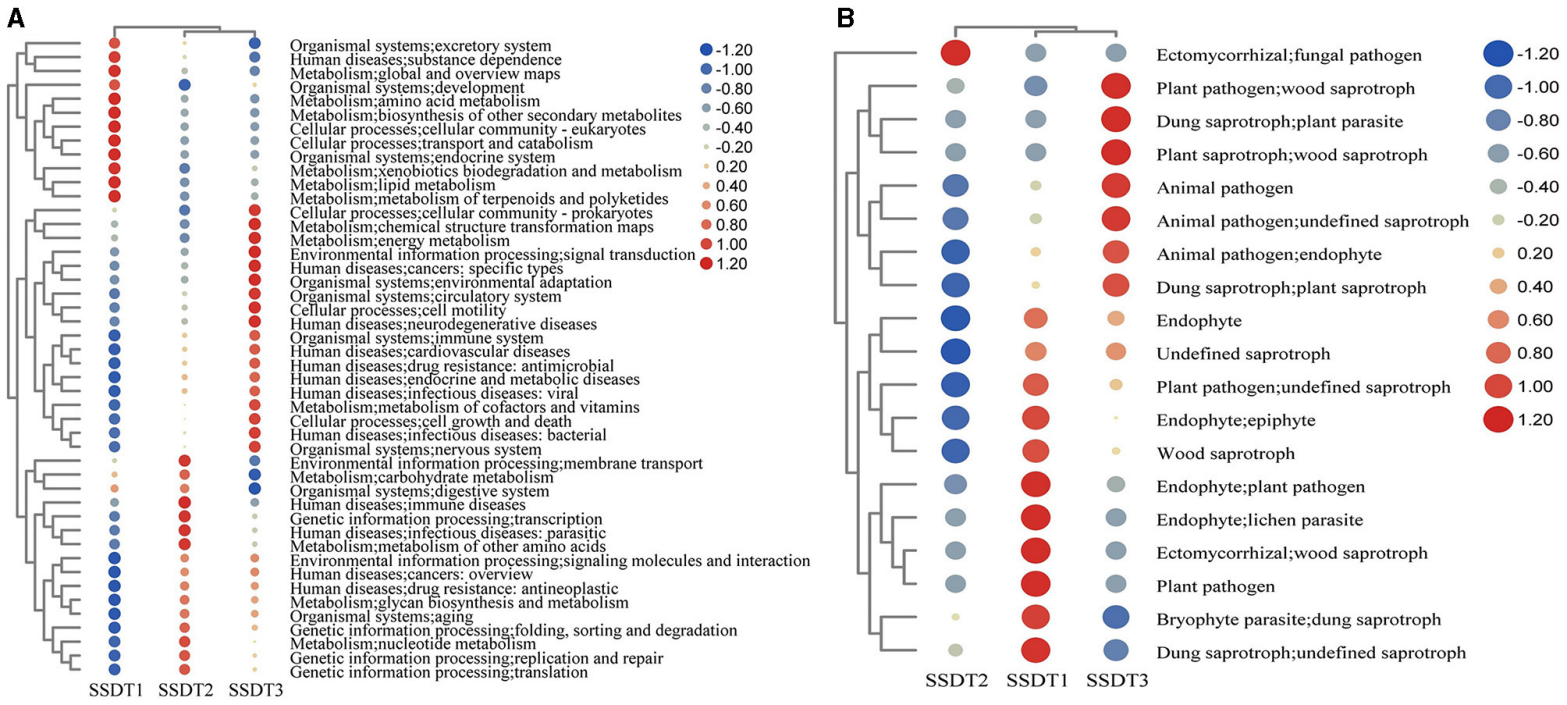
Prediction of microbial function. **(A)** Bacterial function based on the relative abundance of bacterial KEGG prediction using Tax4Fun, the functional abundance changes are reflected by the color gradient of the color bubble with the scale bar showing the variation range of its normalized abundance. **(B)** Fungal function based on FUNGuild.

In contrast, SSDT2 exhibited a higher abundance of functions related to energy metabolism, metabolism of cofactors and vitamins, signal transduction, cell growth and death, and environmental adaptation. Notably, SSDT2 also had more functions related to human diseases, such as specific types of cancer, neurodegenerative diseases, cardiovascular diseases, antimicrobial drug resistance, endocrine and metabolic diseases, and viral and bacterial infectious diseases. In SSDT3, the bacterial community was primarily involved in the metabolism of other amino acids, nucleotide metabolism, carbohydrate metabolism, immune diseases, parasitic infectious diseases, replication and repair/translation of genetic information processing, etc. (Figure 4A).

**Figure 5:**
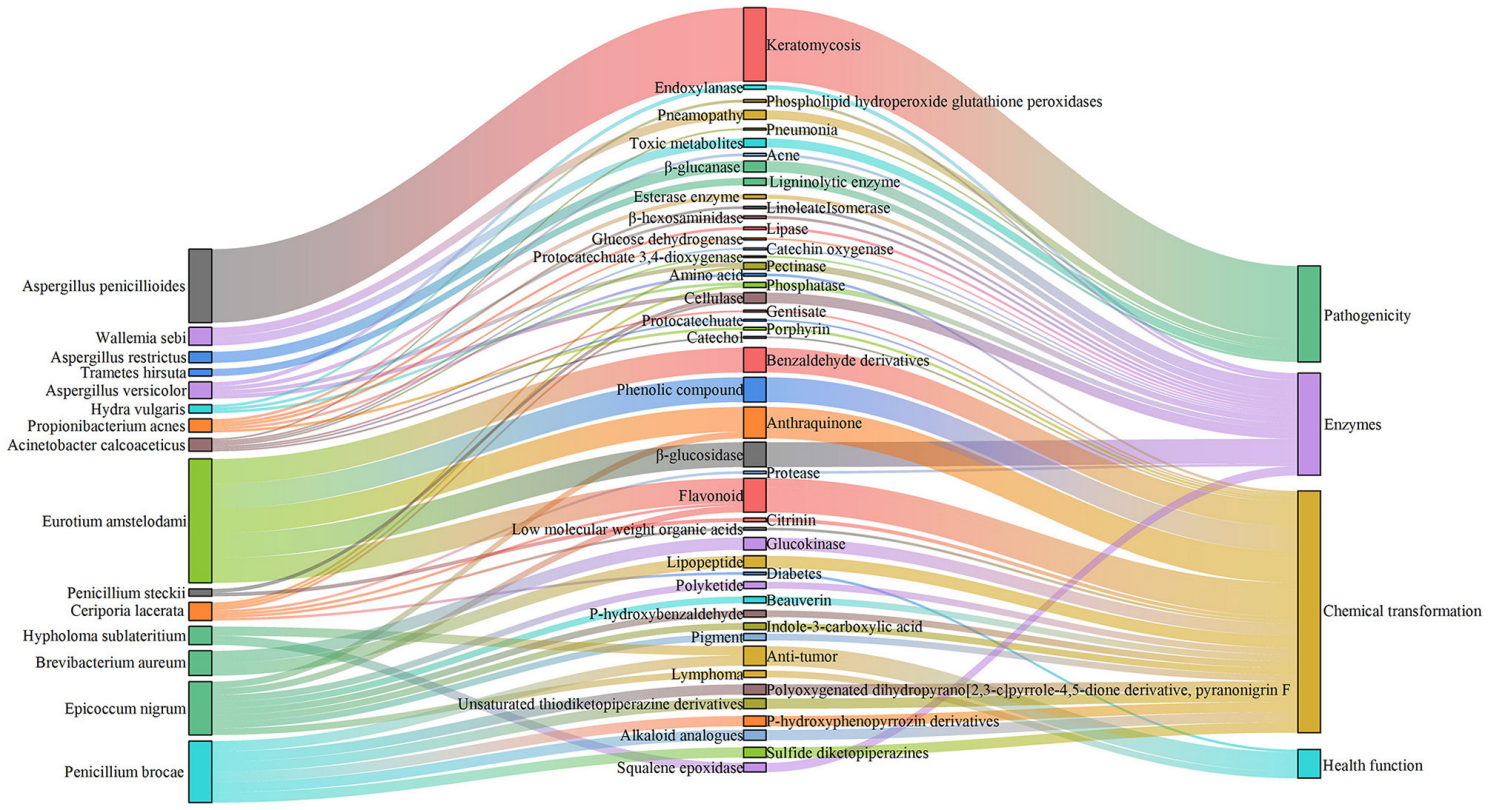
Function of certain airborne microbes with higher abundance.

Simultaneously, FUNGuild analysis showed that in SSDT1, endophyte epiphyte, wood saprotroph, plant pathogen, endophyte lichen parasite, dung saprotroph, etc., were dominant. In SSDT2, the fungal pathogen was dominant. In SSDT3, wood saprotroph, plant parasite, animal pathogen, and plant saprotroph endophyte were abundant (Figure 4B). Further analysis suggested that the airborne microbes with higher abundance appeared to be highly involved in enzyme production, chemical transformation, and health benefits while also exhibiting opportunistic pathogenic characteristics (Liu et al., 2023; Yang et al., 2024; Kavanagh et al., 2018). Notably, certain microbes, such as *Eurotium amstelodami*, are highly associated with the transformation of phenolic compounds, flavonoids, and benzaldehyde derivatives (Gu et al., 2019), highlighting their importance in SSDT production (Figure 5).

## Discussion

This study comprehensively reveals the airborne microbial communities and their functions in a typical SSDTPP during production. The species of bacteria and fungi present in the SSDTPP air were both abundant and diverse, with the microbial communities evolving throughout the pile fermentation process. Indeed, the stability of the microbial community structure was challenging to maintain over time due to unstable conditions in the air, as influenced by factors such as fluctuations in relative humidity, temperature, light, nutrients, and dust (Chen et al., 2020; An et al., 2013; Puspitasari et al., 2016; Ladau and Eloe-Fadrosh, 2019). The water content and temperature of the piled tea decreased and fluctuated during production (Wen et al., 2010), which, combined with changes in SSDTPP air temperature and humidity, contributed to the variation in airborne microbial communities in SSDTPP during pile fermentation.

In this study, numerous microbes that coexisted in both SSDTPP air and piled SSDT were discovered, suggesting that SSDTPP may be a complete microecological environment with a unique microbial cycling system, in which microbes are frequently exchanged between air and piled tea. This exchange appears to be highly related to the “turning of the piled tea,” which is done 3–4 times during pile fermentation to ensure consistent fermentation and promote tea quality. Most airborne microbes are generally loaded by the particulate matter in the atmosphere (Chen et al., 2020), whose natural settling on the piled tea stimulates the fermentation process. During the turning, the microbes inhabiting the piled tea would be spread into the atmosphere by the dispersal of air current (Federici et al., 2018), which could be an appropriate explanation for the co-presence of microbes in SSDTPP air and piled tea.

Additionally, the microbial diversity in SSDTPP air changed similar to that of piled SDT during pile fermentation (Yan et al., 2021a,b). *Saccharopolyspora, Kocuria, Brachybacterium, Burkholderia*, and *Sphingomonas*, observed in both SSDTPP air and piled SSDT, were also detected in piled SDT during production (Zou et al., 2022; Yan et al., 2021b). All these findings suggested that the microbial populations in a specific dark tea production region may be highly similar. Furthermore, the abundant *Penicillium brocae* discovered here was previously detected by Xiong (2017) in the same SSDTPP using the culture method, implying that the microbial species in the same SSDTPP microecological environment may be relatively stable.

*Aspergillus* is mainly involved in dark tea quality development by actively participating in the chemical transformation during pile fermentation (Li et al., 2018; Lin et al., 2021). It could secrete a wide range of enzymes, especially a large number of glycoside hydrolases (GHs), such as amylases, cellulase, β-glucosidases, and xylanases, to promote the decomposition of polysaccharides, thus damaging the tea leaf cell wall and stimulating a series of chemical reactions (Zou et al., 2023b). *Penicillium* appears to be a major source of highly active GHs (Sreeja-Raju et al., 2020; Volkov et al., 2020) and beneficial for dark tea flavor. *Eurotium amstelodami* can produce β-glucosidase and contribute to the flavonoid glycoside conversion (Gu et al., 2019). *Debaryomyces hansenii* is involved in carbohydrate degradation and catechin transformation of SSDT (Zou et al., 2023a). Some *Saccharpolyspora* species exhibit antitumor activity (Liu et al., 2005). Certain *Burkholderia* species can protect crops from fungal diseases (Holmes et al., 1998), while *Kocuria* species can produce antibacterial substances and are involved in transforming polysaccharides (Yan et al., 2021b; Guesmi et al., 2022). *Sphingomonas* has an excellent capacity to degrade both natural and xenobiotic compounds (Asaf et al., 2020). These airborne microbes, especially the highly abundant *Aspergillus* detected here, suggested that SSDTPP air may be a potential reservoir of functional microorganisms. Particularly, some functional microbes contributing to dark tea production, such as *Eurotium amstelodami*, coexisted in piled SSDT and SSDTPP air. This finding suggests that the airborne microbes may also play an important role in SSDT quality development via various microbial enzymes to accelerate tea chemical transformation when exchanged for piled tea, but whether the airborne microbes and piled tea microbes have a competitive or synergistic influence on tea quality requires further research.

It is worth noting that some opportunistic pathogens have been detected in SSDTPP air. For example, *Acinetobacter calcoaceticus* can cause pneumonia (Bilgiç et al., 1995), and *Aspergillus penicillioides* is associated with Keratomycosis (Machowicz-Matejko et al., 2018). Inhalation and dermal contact with these opportunistic pathogens may pose a health risk to workers at the site (Li et al., 2013; Liu et al., 2018).

Some pathogens detected in the SSDTPP air were also found in the piled tea. However, these pathogens are opportunistic and are also commonly present in water, plant rhizospheres, animals, and foods (Wong, 2013). Injections are mainly administered to patients who are immunocompromised or have injuries (Hassan et al., 2024), and such infections are relatively rare from a clinical perspective.

Additionally, the impact of opportunistic pathogens on SSDT quality and potential health hazards requires further research. To date, guidance and information on the health risks associated with airborne microbial exposure in dark tea pile fermentation plants remain limited. Nevertheless, it is still recommended that strict protective measures, such as wearing special work clothes and protective goggles, be employed in SSDTPPs to reduce the risk of infection.

## Conclusion

Microorganisms play a crucial role in shaping the quality of SSDT, with the air in the SSDTPP considered the primary source of these microbes. In this study, during pile fermentation, we identified a total of two phyla, nine classes, 18 orders, 28 families, 38 genera, and 47 species of fungi, alongside 24 phyla, 49 classes, 88 orders, 153 families, 253 genera, and 90 species of bacteria in the SSDTPP air. The highest fungal and bacterial diversities were detected in SSDT1 air and SSDT2. *Aspergillus* was remarkably dominant in SSDTPP air, followed by *Saccharopolyspora, Penicillium, Peseudonocardia, Epicoccum, Eurotium, Staphylococcus*, etc.

The airborne microbes in SSDTPP may frequently exchange with those inhabiting the piled tea, as numerous microbes have been found to coexist highly in both environments, the air and the piled tea. These airborne microbes may be significantly involved in enzyme production, chemical conversion, and health benefits while also having the potential to act as opportunistic pathogens. These results pave the way for microbial traceability in piled SSDT during production and significantly advance the understanding of microbial influences on SSDT quality.

## Data Availability

The datasets presented in this study can be found in online repositories. The names of the repository/repositories and accession number(s) can be found at: https://www.ncbi.nlm.nih.gov/, PRJNA1103387.

## Supplementary material

Supplementary Figure S1 Sichuan south-road dark tea.

Supplementary Figure S2 Sequencing profile **(A)** Length distribution for fungal sequencing. **(B)** Length distribution for bacterial sequencing. **(C)** Rank-abundance curves of fungal OTUs. **(D)** Rank-abundance curves of bacterial OTUs.

Supplementary Table S1 Sequencing data profile of airborne microbes in SSDTPP during pile fermentation.
