# Supplementary figures and images for: Insights into the airborne microorganisms in a Sichuan south-road dark tea pile fermentation plant during production
Source: Front Microbiol. 2024 Sep 2;15:1439133. doi: 10.3389/fmicb.2024.1439133 (PMC11402737; doi:10.3389/fmicb.2024.1439133)

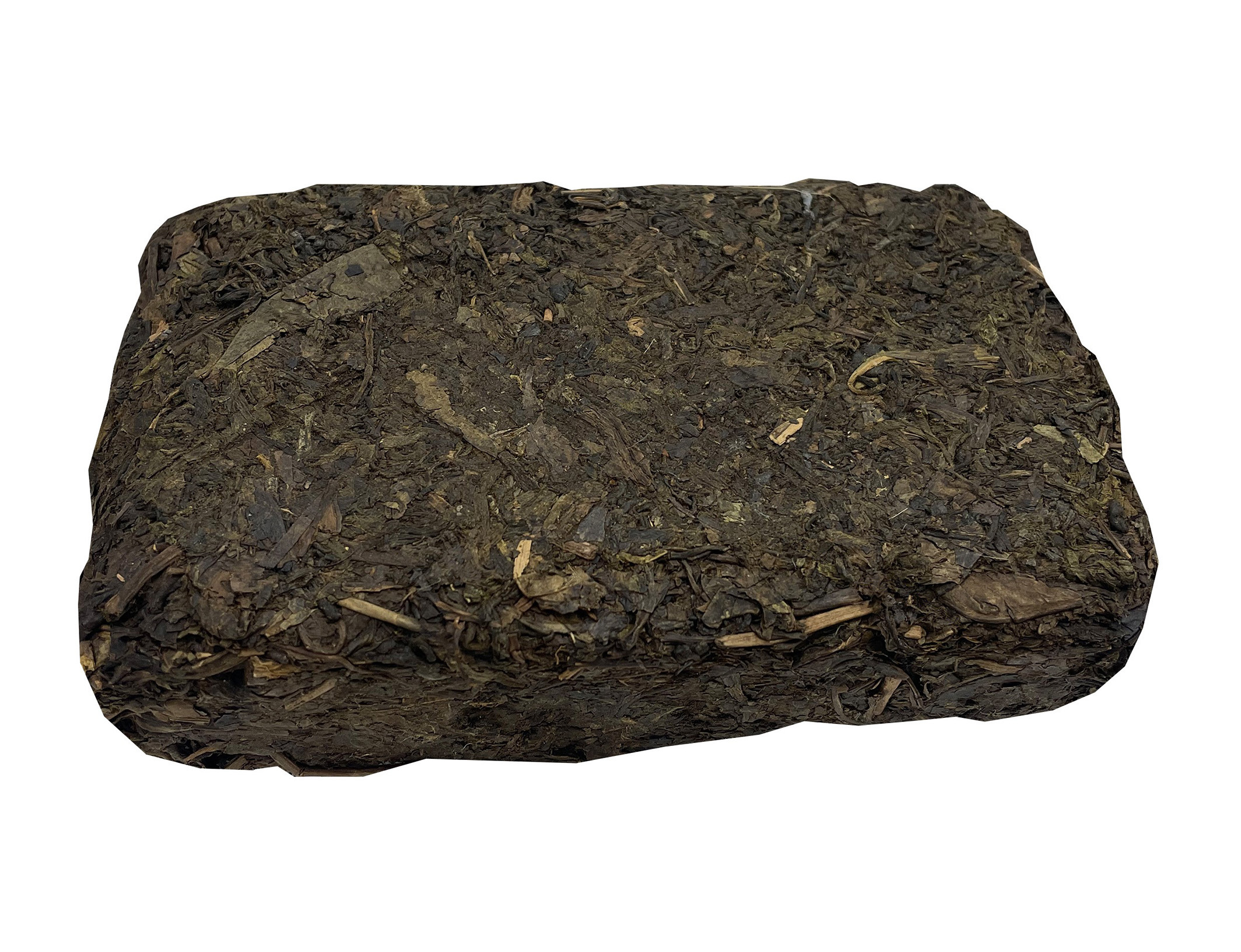

Supplement: Supplementary Figure S1 — Sichuan south-road dark tea. [file Image_1.TIF]

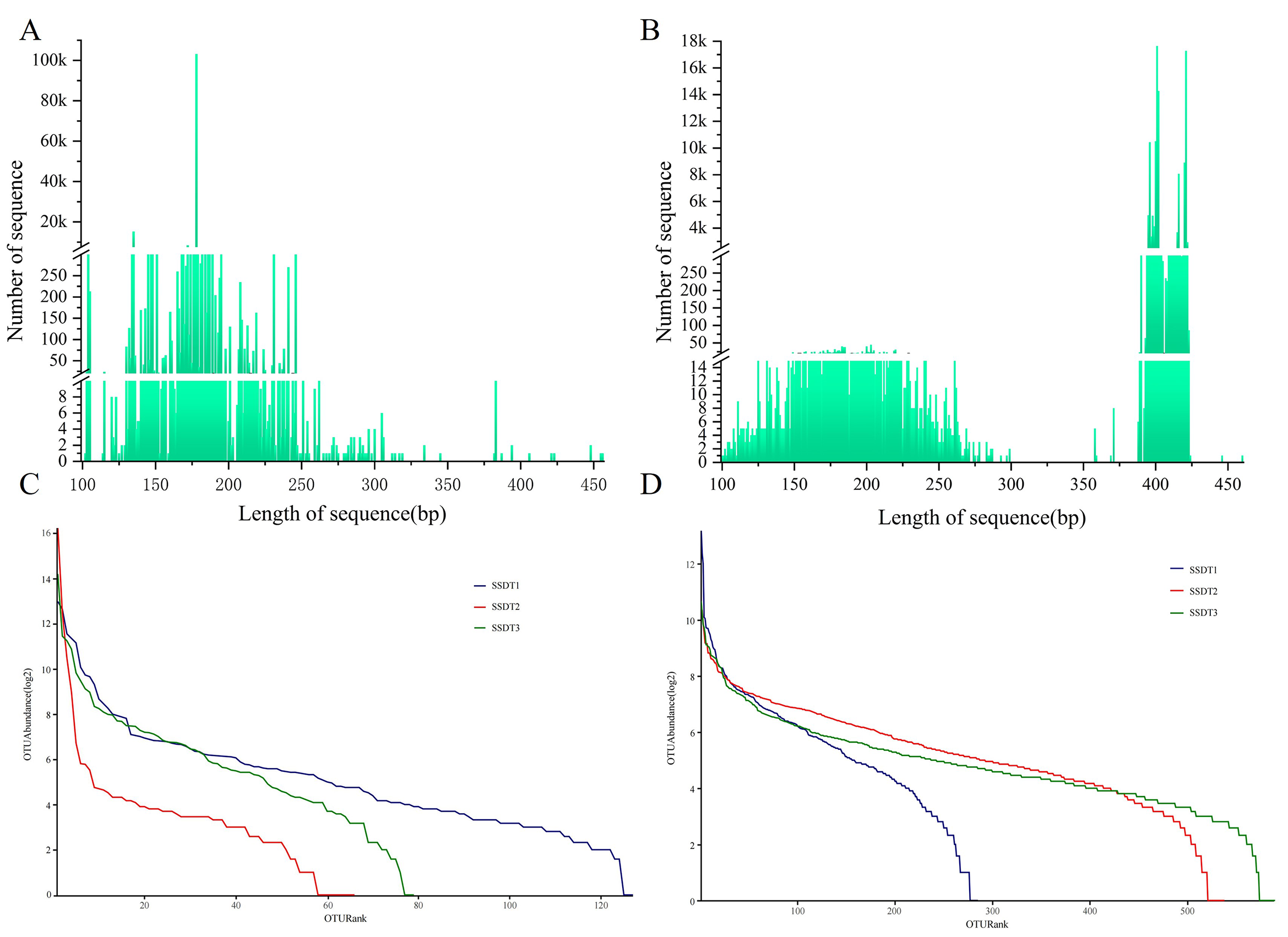

Supplement: Supplementary Figure S2 — Sequencing profile (A) Length distribution for fungal sequencing. (B) Length distribution for bacterial sequencing. (C) Rank-abundance curves of fungal OTUs. (D) Rank-abundance curves of bacterial OTUs. [file Image_2.TIF]
