# Supplementary material for: Insights into the airborne microorganisms in a Sichuan south-road dark tea pile fermentation plant during production
Source: Front Microbiol. 2024 Sep 2;15:1439133. doi: 10.3389/fmicb.2024.1439133 (PMC11402737; doi:10.3389/fmicb.2024.1439133)
Supplement: Supplementary Table S1 — Sequencing data profile of airborne microbes in SSDTPP during pile fermentation. [file Table_1.DOCX]

**TABLE S1 |** Sequencing data profile of airborne microbes in SSDTPP during pile fermentation

| Sample | Valid Count | | Good Count | | Percentage | |
| --- | --- | --- | --- | --- | --- | --- |
|  | Fungi | Bacterium | Fungi | Bacterium | Fungi | Bacterium |
| SSDT1 | 32069 | 47457 | 31833 | 46136 | 99.26% | 97.22% |
| SSDT2 | 33909 | 41370 | 33724 | 39990 | 99.45% | 96.66% |
| SSDT3 | 89005 | 35841 | 88474 | 34338 | 99.4% | 95.81% |

*“Valid Count” represents the number of valid sequences, “Good Count” represents the number of*

*high-quality sequences, and “Percentage” represents the proportion of high-quality sequences to*

*effective sequences.*
